# Pet Presence Can Reduce Anxiety in the Elderly: The Italian Experience during COVID-19 Lockdown Assessed by an Electronic Survey

**DOI:** 10.3390/ijerph19106135

**Published:** 2022-05-18

**Authors:** Daniele Giansanti, Mariacristina Siotto, Laura Parisi, Irene Aprile

**Affiliations:** 1 Istituto Superiore di Sanità, 00161 Rome, Italy; daniele.giansanti@iss.it (D.G.); laura.parisi57@hotmail.com (L.P.); 2 IRCCS Fondazione Don Carlo Gnocchi, 50143 Firenze, Italy; msiotto@dongnocchi.it

**Keywords:** COVID-19, social distance, elderly, anxiety, pet, mental-health

## Abstract

The lockdown imposed in Italy due to the COVID-19 outbreak required restrictions that severely limited individual freedom to protect the population and reduce virus diffusion. This situation psychologically challenged the entire Italian population but mostly the elderly. The “Digital mental health approach” employs digital tools to evaluate and prevent increasing mental health problems. “Anonymous online electronic surveys” are digital tools that assess rates of mental health outcomes (using for example self-assessment/awareness tools). Immediately at the beginning of restrictions, we designed an electronic survey a) to remotely investigate the psychological impact of the lockdown and b) to compare the anxiety between pet owners and not-pet owners. A total of 3905 subjects filled out the survey; we focused our study on 781 (20%) elderly subjects. Dividing elderly patients between pet-owners (*n* = 405) and not-pet owners (*n* = 376), the pet owners showed a Zung scale score significantly lower in respect to the not-pet owners. We observed that, during the COVID-19 outbreak, the pet presence could have a positive effect on anxiety in the elderly subject. These results: (A) encourage the use of mobile technologies for the assessment of psychological disorders that can be promptly employed in emergencies such as the COVID-19 outbreak; (B) highlight the positive effect of pet interaction to mitigate the psychological distress in elderly people.

## 1. Introduction

On 11 February 2020, the WHO announced the name of the respiratory disease caused by the new coronavirus: COVID-19. On 9 March 2020, in response to the growing pandemic of COVID-19 in the country, the Italian government imposed lockdown measures, including movement restrictions (with some emergency exceptions). The restrictions aimed to protect the population and reduce virus diffusion, severely limiting individual freedom. The citizens, for the first time, experimented with an imposed reclusion. There was a concern about the lockdown’s effects on the psychological health of elderly people because of loneliness and isolation that could have exacerbated their mental health conditions [1]. Indeed, the most common mental and neurological disorders in people over 60 years are dementia and depression, which affect approximately 5% and 7% of the world’s older population, respectively while anxiety disorders affect 3.8% of the older population [2]. The technological tools were essential in supporting people during the restrictions imposed by COVID-19 [3,4]. Technological solutions were useful for maintaining social contacts, thanks to video-call software and/or messaging Apps. These valuable tools include easy and friendly solutions-based, for example, mobile technology, thanks to which a large number of people could be reached also in unexpected situations [5,6] such as the current outbreak of COVID-19, which require social distancing. In this context, electronic health and mobile health are the two core elements [7] for the interventions in digital health. Digital health interventions have frequently been highlighted as one way to respond to increasing levels of mental health problems, for example, in children and young people [8]. Moreover, these interventions can be also addressed for the prevention and early intervention of mental health problems thanks to tools like online electronic surveys (eSs) [8,9]. eSs assess rates of mental health outcomes (e.g., self-assessment/awareness tools based on consolidated psychological tests), thus supporting structured mental health interventions which can contrast some negative effects of different social issues. Therefore, eSs appeared very useful to detect mental health problems caused by the COVID-19 outbreak, both in general and in at-risk populations. ESs enabled the remote administration of psychological tests through electronic tools with modern systems based on mobile technology. This approach showed two potentialities: the first is reaching subjects and investigating their state of mental health, even for preventive purposes; the second consists in setting up a self-awareness tool that stimulates subjects to take care of themselves [8,9].

New approaches to mitigate psychological distress are based on the use of pets. This approach is commonly known as animal-assisted therapy (AAT). In general, AAT is an alternative or complementary therapy that includes the use of animals (horses, dogs, and other domestic pets like cats and rabbits). It consists of activities that range from co-presence to more targeted ones.

A Pubmed search with the composite key: *(animal assisted therapy [Title/Abstract]) AND ((anxiety [Title/Abstract]) OR (depression [Title/Abstract]) OR (mental health [Title/Abstract]) OR (psychological disorders [Title/Abstract]) OR (psychological distress [Title/Abstract]))* reports that to date there are 34 reviews [10] that make a map point on this issue under different perspectives, conditions, situations and types of participants.

The study by Bolding and Butala [11] faced the application of AAT in neurological disorders such as stroke, dementia, Parkinson’s disease, multiple sclerosis, Huntington’s disease, epilepsy, and acute brain injury. Their review examined the effects that emotional support dogs, dog therapy, or dog ownership has on these specific neurological disorders. Dog therapy and ownership were found to improve mood, quality of life, and disease symptoms across multiple neurological disorders.

The report by Young and Horton [12] focused on the AAT applied to post-traumatic stress disorders, anxiety disorders (including generalized anxiety disorder), and major depressive disorders. Treatment strategies for these psychological disorders often include clinical care with pharmacologic agents (e.g., antidepressants), psychotherapy, or a combination of both approaches. The study summarized and appraised the available evidence on the clinical effectiveness of canine therapy and equine therapy versus the other traditional approaches.

The meta-analysis by Ein et al. [13] examined the efficacy of the AAT as a method for reducing physiological stress levels (blood pressure and heart rate) and subjective stress and anxiety scores (self-reported stress/anxiety). Using a random effects model, they determined that significant differences occurred in heart rate, self-reported anxiety, and self-reported stress after AAT exposure compared with before AAT. Also, the study by Cherniak et al. [14] investigated this specifically in older adults [14]. AAT has been shown to improve social, physiological, and psychological patient performances. The positive effects of AAT were investigated by means of subjective self-assessment scales and by means of objective physiological measures—such as pressure blood, heart rate, cholesterol levels, hormone levels, and other parameters [14]. The brief overview, based on targeted reviews, shows how there are many psychological aspects to be investigated regarding the impact of the AAT. These include mood, quality of life [11], post-traumatic stress disorders, anxiety disorders (including generalized anxiety disorder), major depressive disorder, and other forms of psychological distress [12]. Each of these psychological distress deserves and needs targeted approaches with a specific experimental set-up. There are also some basic problems with the relationship between some of these forms of psychological distress, currently the subject of studies [15]. Also, from this emerges the need to address, in an innovative approach such as AAT, one issue at a time.

The mechanism which explains the positive effect of the owner-pet interaction is currently under study; however, some studies suggested that these positive effects can depend on (a) the encouragement to do physical activity while walking the dog and (b) the co-presence of a pet [14,16,17].

The COVID-19 outbreak seriously limited movements; the only physical activities allowed outside were: purchasing food or essential goods close to home or walking the dog. On this basis, we supposed that the interaction with the pet, based on the co-presence and/or the walking together, could have a positive effect on the emotional sphere of the elderly people during lockdown for COVID-19 and, in light of the above, focused on one of the forms of psychological distress: the anxiety. Therefore, we designed a specific electronic tool (eS) to collect information about: (I) pet ownerships, (II) the type of interaction with the pet (for AAT investigation); and (III) anxiety. To include also subjects less accustomed to digital technology, we spread it through social media, with peer-to-peer techniques. The eS was administered in the restricted period of lockdown. When the data collection was completed, we analyzed the anxiety levels in elderly populations divided between pet owners and non-pet owners.

## 2. Methods

The self-assessment electronic tool, based on an ad hoc electronic survey, has been designed using Microsoft Forms. The tool included three sections: (a) a section with an informed consent form about the aim of the survey, information related to demographic data (sex, age, school level, country), and the presence or absence of previous psychological problems (subjects with previous mental problems were excluded); (b) a section dedicated to the interaction with the pet (only for pet owners); (c) an optional section containing the anxiety self-assessment scale (SAS) [18]).

Filling in the questionnaire was anonymous and optional. We have foreseen a peer-to-peer diffusion through social media, encouraging those most familiar with technology to support the less accustomed. Circumstances never experienced and the kind of scientific exploration without previous references suggested us not to put age constraints on inclusion. The only inclusion constraint was to include only subjects who have never had psychological problems. The inclusion of subjects with psychological problems would have required a more articulated study configuration path also from the point of view of the formal approval, incompatible with the rapid start-up times of a few days. The eS is available at the link reported in [19].

The section dedicated to the pet owner comprises dog and cat owners, including: (a) Questions about pet ownership, the number of pets owned, and if the pet lives in the house. (b) A Likert (question 16) with a set of statements, each with 6 levels of evaluation, ranging from a minimum of 1 to a maximum of 6 (1 = not at all agree with the statement; 6 = completely agree with the statement). An answer greater than 3.5 indicates a value of more yes than no; while a value below 3.5 indicates a value of more no than yes. (c) A free open question about the sensations that the animal generates. Appendix A shows an example of a piece of the eS dedicated to the ownership and interaction with the dog. Question 13 with a yes/no option dedicates to dog ownership. If the interviewee participant owns the dog, a further menu opens with other questions. Question 14 asks for the number of dogs. Question 15 asks if the animal lives at home. Question 16 is the Likert quoted above with a series of statements. Question 17 is an open question.

A section of the eS, reported in Appendix B, allows you to acquire demographic data. Question 2, a choice question, asks if you have had any previous psychological problems. Questions 3 and 4 are choice questions and require gender and age. Questions 5 and 6 are choice questions and are dedicated to students. Question 7 is a choice question and requires a degree of training. Question 8 is an open question and requires information about the work situation (e.g., I do not work, I work as…, I am retired, I am on layoffs). Question 9 asks for information on how the lockdown is being experienced, asking for information describing how you are living the lockdown (e.g., family, activity, social and environmental interactions). Question 10 requires indicating the region, province, and municipality where you are living during the lockdown. Question 11 asks if you are living in a red zone. An area with restrictions during the pandemic.

All these questions relating to the acquisition of demographic data had as a first basic control, a mandatory response check (the submission of the form does not close if the participant does not answer the mandatory answers).

Question 2 sends, in the event of an affirmative answer (presence of previous psychological problems and therefore exclusion from the survey), through a branching control, to a final comment based on an open answer. The choice questions, with two or multiple choices, have a simple control and allow an automatic categorization. Open questions have a more complex control as they must be examined one by one for extraction of the contents for a further thematic analysis

This approach allows, through scalable mechanisms and incremental data mining, to carry out targeted analyses, of which the one reported in this work represents the first step.

The section dedicated to the anxiety evaluation included a self-assessment scale (SAS) [13], which measures the current psychological state of subjects. Subjects were asked to read carefully 20 sentences listed and to choose the answer that better describes the current subject’s feeling. For the SAS, the cut-off point recommended by Zung is a raw score of 36, but we used a raw score of 40 which is considered most appropriate in research focused on individuals living in the community [18,20]. The developers submitted the eS through social networks (e.g., Facebook, Twitter, Linkedin) and messaging tools (e.g., Messenger, WhatsApp) which allowed a peer-to-peer diffusion. We invited the most accustomed to technology subjects to involve the less practical ones, in order to minimize bias and limit the impact of the digital divide. Appendix B reports the details of this invite included in the eS itself, as well as in the text accompanying the internet link. The Quick Response code associated with the eS was also obtained from the link. Thanks to this image, as it is known, it is possible from a smartphone or tablet to open the survey and fill it in, simply by pointing to a paper print of this image. This guarantees an additional channel of diffusion. Appendix B also reports this Quick response code. The eS was sent on March 15, subjects began responding on March 16 exactly one week after the start of the lockdown in Italy (which occurred on 9 March 2020). The eS remained active until March 25.

## 3. Results

We sent 4993 eSs: 389 subjects did not give the consent, 799 subjects did not fill in the anxiety test, and 3905 anonymous subjects with and without a pet (dog and/or cat) filled out the survey completely (age: 14–77 years; average age 44.7 years; 1913 males; 1992 females). Elderly subjects (≥65 years) were 781 (20%) of the entire group. 83.73% of responses were obtained within 2 days, while the 98.66% within 6 days. This study was focused on the elderly population; we found that 40.3% of the elderly subjects showed a Zung scale score ≥ 40 during the lockdown. We divided elderly participants (aged between 65 and 77 years) between pet-owners and not-pet owners (Table 1).

The pet owners showed a lower averaged Zung scale score. The difference between, respectively, the mean Zung scale score in the group of not pet owners and the mean Zung scale score in the group of pet owners was ∆m = +8.0. We applied a statistical approach based on both the χ^2^ test and the Student *t*-test.

We first applied to the sample a statistical approach based on the χ^2^ to test the difference in frequencies. We applied the two dimensions χ^2^ test with the two variables (pet owners, not pet owners) and (Zung scale score ≥ 40; Zung scale score < 40). The first variable controls the therapy (AAT), and the second measures the outcome using the Zung scale score according to the threshold. The χ^2^ test returned a high value of significance (*p* << 0.01) in the difference in frequency of the Zung scale score ≥ 40 between the groups. We further investigated, using the frequency analysis, whether and how the decrease in anxiety was affected by their relationship with the pet (Appendix A, Likert, Question 16). All subjects in the group of pet owners returned a response with a value greater than 3.5 (corresponding to more yes than no) for three statements: (i) having it in the same room; (ii) playing with it; (iii) taking care of it. Each of the other 3 statements (sleeping with it; walking with it; seeing it walk out of) was identified in two groups: (a) responses with an assessment > 3.5 (corresponding to more yes than no); (b) responses with an assessment <3.5 (corresponding to more no than yes). The χ^2^ test showed that in these 6 groups there was no statistical difference among the measure of anxiety detected [χ^2^ (5, *n* = 405) = 1.01, *p* = 0.96].

We investigated the hypothesis of normal distribution for the two groups concerning both the age and the Zung scale score. We tested the normal distribution of age by the Smirnov–Kolmogorov test of normality, which is suitable for large samples such as ours.

The sample of 405 elderly pet owners had a normal distribution in relation to age. The null hypothesis was that our data follows a normal distribution. We achieved *p* = 0.51. Because *p* > 0.05, we accepted the null hypothesis. We are therefore facing a normal distribution. The sample of 376 elderly, not pet owners had a normal distribution in relation to age. The null hypothesis was that our data follows a normal distribution. We achieved *p* = 0.53. Because *p* > 0.05, we accepted the null hypothesis. We are therefore facing a normal distribution.

The sample of 405 elderly pet owners had a normal distribution in relation to the Zung Scale score. The null hypothesis was that our data follows a normal distribution. We achieved *p* = 0.54. Because *p* > 0.05, we accepted the null hypothesis. We are therefore facing a normal distribution.

The sample of 376 elderly, not pet owners had a normal distribution in relation to the Zung scale score. The null hypothesis was that our data follows a normal distribution. We achieved *p* = 0.55. Because *p* > 0.05, we accepted the null hypothesis. We are therefore facing a normal distribution.

Based on the outcome confirming the normal distribution we applied a statistical approach based on the differences among the averaged Zung scale score. We applied *the* Student *t*-test. The tested hypothesis was the significance of the difference in the means of the Zung scale score, between the group of 405 elderly pet owners and 376 elderly, not pet owners. We therefore applied the Student *t*-test between the mean Zung scale score in the group of not pet owners and the mean Zung scale score in the group of pet owners showing a difference ∆m = +8.0. The Student *t*-test returned a high significant value (*p* = 0.008 < 0.01) of the difference between the averaged Zung scale score in the two groups.

## 4. Discussion

There is no doubt that the first and true lock-down period, imposed by the COVID-19 pandemic, has highlighted the extreme vulnerability of the elderly people, due to their greater degree of fragility than other people have. On the other hand, the inability in the first period to support the frail people with adequate remote services was also highlighted [21] and the need to focus on them in the subsequent phases was recalled [22]. The eSs developed in this study join the analysis of the pet-owner relationship in relation to anxiety and the advantages derived from digital health [8,9]. Several studies have shown that pets are valid support during strong emotional stress [23,24,25,26]. However, it has been reported the importance of objectively quantifying such a positive pet-owner interaction [16,17] to overcome emotional distress [27]. The eS developed and presented herein has been promptly submitted through modern communication Apps, to 4993 subjects, receiving almost all responses within 6 days. Thus, showing the efficacy and speed given by these new digital technologies. This study was designed to focus only on elderly subjects (*n* = 781), with the aim to evaluate the anxiety and the impact of the pet’s presence on the anxiety.

The first important result of this survey is the very high anxiety prevalence rate among the elderly during the COVID-19 outbreak (40.3%). Before the COVID-19 pandemic, the prevalence rate of anxiety disorders was 1.2–15% in older individuals living in community samples and 1–28% in individuals hospitalized [28]. WHO reported a prevalence of 3.8% of anxiety disorders in the elderly population [2]. Therefore, the COVID-19 outbreak caused an increase of elderly subjects who complained of mental problems such as anxiety with a percentage ten times higher than those previously observed. This highlights that the elderly population appeared to be particularly vulnerable during the first, unexpected periods of lockdown in Italy. This can be addressed, both to the severity of the restrictions and to the lack of the presence of remote services capable of supporting frail people, especially elder ones [21]. Studies conducted in China, where the pandemic first exploded, revealed similar results [29]. After more than one year the scenario of psychic discomforts seems changed: the prolonged restriction period caused mental distress, especially in the younger people, and several studies were focused on them to propose valid solutions [30]. Nevertheless, our results, compared to other studies, emphasize the strong need to focus on the elderly population considering its vulnerability and frail mental health [31,32].

The second important and central result is the impact of the pet on anxiety. The outcome highlighted the higher anxiety prevalence among elderly that were not-pet owners with respect to pet owners. Therefore, it seems that the lockdown imposed by the COVID-19 pandemic increased the anxiety levels among elderly people, while in the meantime it revealed the positive effect of the pet presence among the elderly.

This central result is in line with the need to quantify the impact of AAT as an alternative therapy for the prevention of psychological problems. Often, even if the AAT is recognized as having a therapeutic value, it is difficult to quantitatively assess its potential and influence [21,22]. This study made it possible to obtain objective quantitative data, on the measure of anxiety in elderly subjects, with and without the use of this pet-based therapy. From a general point of view, it is in line with the approaches in the literature that use the AAT on the elderly [33], as evidenced by a specific search on Pubmed with a composite key:

Search: *(animal assisted therapy [Title/Abstract]) AND (elderly [Title/abstract]) AND ((anxiety [Title/Abstract]) OR (depression [Title/Abstract]) OR (mental health [Title/Abstract]) OR (psychological disorders [Title/Abstract])) Filters: Review*

Which reports four interesting reviews that highlight a positive impact of AAT on minimizing anxiety on subjects with and without neurological diseases [14,34], with minimal risk [35] and with potential also on other psychological problems such as depression [36].

The results are also in line with other studies focused on the advantages of AAT when there are important situations of constraint [37] that in a certain sense are similar to lock-down. The study by Villafaina-Domínguez [37] reviewed the ATT, applied in concrete dog-assisted intervention introduced in prisons to reduce recidivism as well as to improve the well-being of prisoners. The vast majority of protocols included activities related to dog training, dog caring, or activities, which included vocational or educational components. The study concluded that dog-based animal-assisted therapy may improve anxiety, stress, recidivism, and other social variables in male or female inmates.

The study also has side results related to the combination of digital health to investigate both the anxiety and the impact of the AAT.

The side result is the ability of the methodology based on an eS to reach a big number of people by obtaining a quantitative evaluation of the psychological effect of the pet presence among the elderly. During this outbreak, emerged the importance of digital health as a new method to create psychological defense networks [26]. These networks can be based on group therapy using group communication technology tools [4] adapted for elderly subjects or employing other automatic tools that allow self-therapy [38]. Our study, using digital health, allowed us to identify how the pet presence can represent a psychological defense strategy, even if not exclusive, at least complementary to others. This encourages the use the digital health for the assessment and rehabilitation of psychological disorders that can arise in all the situations of impediment or social block, as this period of COVID-19 pandemic [39], confirming the importance of the technologies as mitigating tools in Italy as in the other parts of the World [40,41].

### Limitations and Future Works

Further targeted insights will give us further information. These insights may also consider the open questions proposed, which we have decided to include such as the question “Enter here information describing how you are living the lockdown (e.g., family, activity, social and environmental interactions)”. Other insights may be dedicated to other categories of participants such as the youngest or adults and to the investigation of the other parameters provided by the eS by means of an incremental datamining approach, also considering the thematic contents provided by the open questions. Furthermore, this study was based on the Zung test applied only one time (one-shot) after a week after the traumatic event (the lockdown). Results, suggest, as further direction: (a) to follow different methods with other tests to be administered with a multi-phase approach to monitoring at predetermined time distances from the traumatic event: (b) to focus, with the specific investigation to all the possible psychological distress, as for example, the depression [36]. On the other hand, an expected limitation of this new approach is represented by the digital divide which can affect some categories of subjects, especially the elderly [40]. However, a piece of correct and punctual information given during the peer-to-peer administration (see Appendix B), conceived to support and involve the less accustomed to technology, allowed to overcome this limitation. The high participation obtained from the elderly population highlights that: (i) if correctly supported, elderly people are able to approach digital technologies; (ii) the results obtained depend also on intergenerational solidarity that allowed a positive approach to new technologies, especially to mobile devices [42]. Another limitation of this study is that we considered all pets without distinguishing between cats and dogs; this should be better analyzed in further studies, to individuate eventual differences due to different relationships and/or daily habits (e.g., walking for a dog could be an extra motivation for minor anxiety).

## 5. Conclusions

The multimedia tools have been proved to be of great support to mitigate the psychological impact of the COVID-19 pandemic and lockdown measures in the Italian general population. The study: (a) demonstrated the usefulness of these tools to investigate the anxiety in the elderly and the impact of the AAT; (b) the importance to invest energy in the AAT that may represent a concrete solution in emergencies, such as the imposed lockdown for the COVID-19 pandemic, to mitigating the psychological distress.

## Figures and Tables

**Table 1:** Details on pet ownership in the elderly (* this number included both 235, not owners of pets and 141 owners of pets who do not live in the house).

Participants	Males (%; Mean Age)	Females (%; Mean Age)	Owners of Pets Living in the House	Presence of One or More Dogs	Presence of One or More Cats	Presence of Cats and Dogs
405	50.7%; 71.7 years.	49.3%; 71.4 years.	yes	189	146	70
376 *	49.1%; 71.2 years.	50.9%; 70.9 years.	no	0	0	0

**Figure A1:**
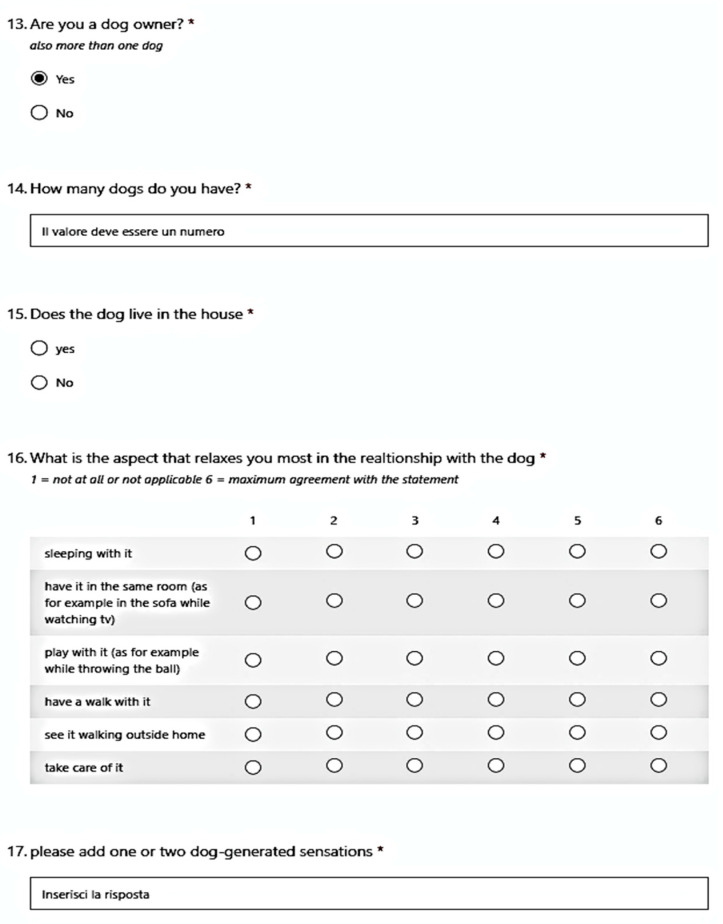
Tool Section dedicated to the questions about the interaction with the dog. “*” means mandatory questions.

**Figure A2:**
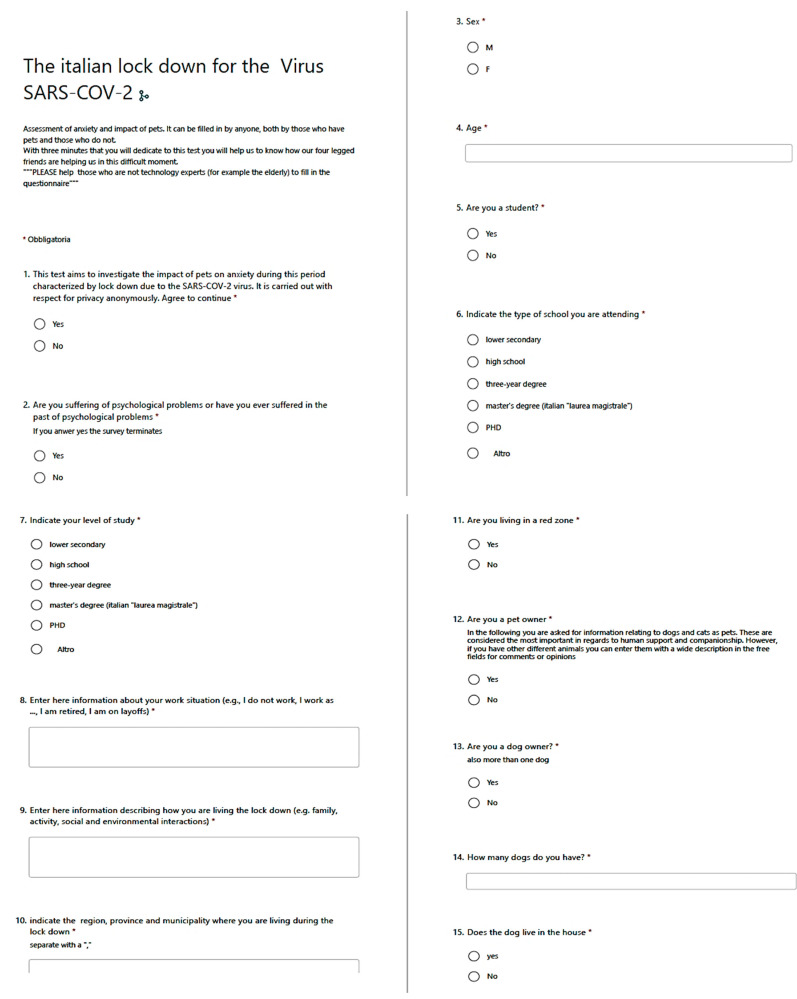
Tool Section dedicated to the collection of the demographic data. “*” indicates mandatory questions.

**Figure A3:**
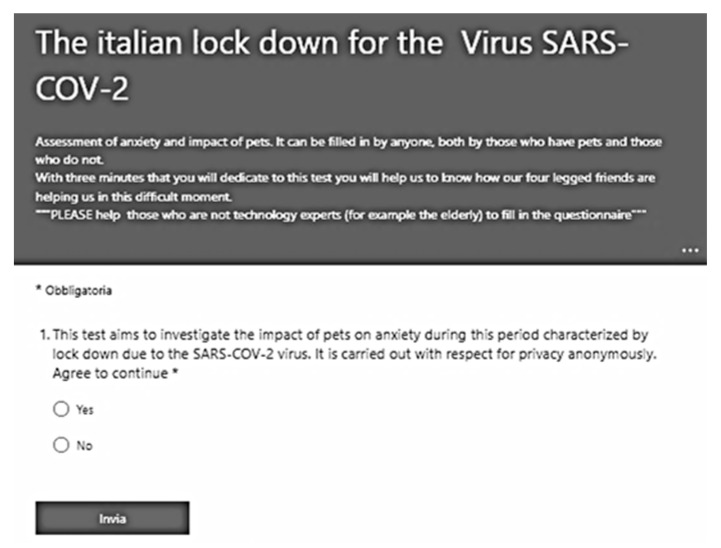
Introduction of the eS with the invite to help those who are not technology experts. “*” indicates mandatory questions.

**Figure A4:**
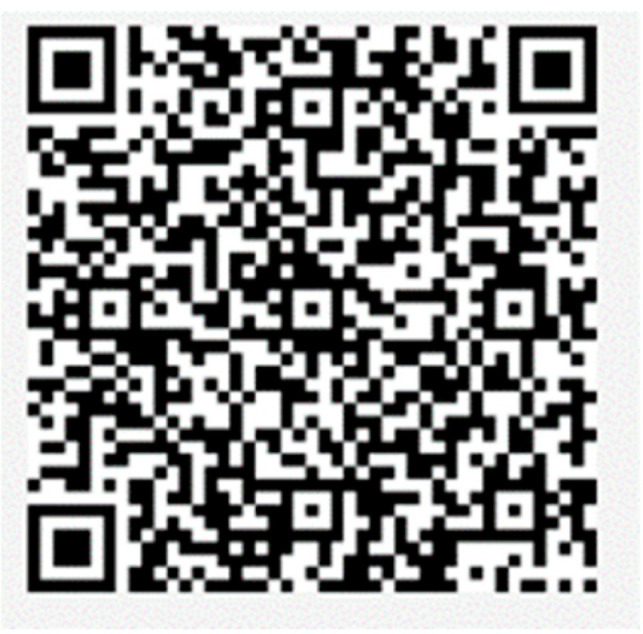
The QR-CODE of the eS.
